# Chiropractic Care in a Patient With Hereditary Spastic Paraplegia and Chronic Pain

**DOI:** 10.7759/cureus.36602

**Published:** 2023-03-23

**Authors:** Eric Chun-Pu Chu, Kevin Hsu Kai Huang, Kenny Cheng, Valerie Kok Yan Chu, Andy Lin

**Affiliations:** 1 New York Medical Group (NYMG) Chiropractic Department, EC Healthcare, Hong Kong, HKG

**Keywords:** genetic analysis, chiropractic management, chiropractic therapy, hereditary spastic pareparesis, rare genetic disease

## Abstract

Hereditary spastic paraplegia (HSP) is a rare neurodegenerative disorder, which is challenging to diagnose and is known to be associated with more than 73 genes. Neurodegenerative disorders are characterized by progressive spasticity and weakness of the lower limbs. Here, we report the case of a 13-year-old girl with a history of HSP who presented to a chiropractic clinic with chronic low back pain and rehabilitation for lower extremity weakness. She had been receiving non-steroidal anti-inflammatory drugs and baclofen for spasticity. Full-spine radiography revealed borderline acetabular dysplasia in the right hip. After nine months of chiropractic therapy, the patient reported reduced lower extremity spasticity and pain as well as improved strength and functionality. As non-invasive therapies have minimal side effects, chiropractic therapy can be used alongside or in combination with other treatments as an additional option for the long-term management of HSP.

## Introduction

Hereditary spastic paraplegia (HSP) is a rare neurodegenerative disorder with an estimated prevalence of 0.1-9.6 per 100,000 people [1]. The wide range in the prevalence of HSP reflects the challenges in diagnosis and the lack of large epidemiological studies. HSP is a diverse disorder, with more than 73 causal genes identified worldwide [2], although some genetic forms are considered to be more common in specific populations [3]. For example, the SPG4 subtype that occurs due to spastin gene mutations may be most prevalent in Europeans, whereas the SPG3A subtype that occurs due to atlastin gene mutations may be more common in Asia [4]. As more HSP genes and variants are discovered, a more accurate understanding of their prevalence and distribution may emerge.

HSP is also a group of neurodegenerative disorders characterized by progressive spasticity and weakness of the lower limbs [5]. The primary pathology is the degeneration of the corticospinal tracts that carry motor signals from the brain to the spinal cord and muscles [5]. This leads to increased muscle tone (spasticity), weakness, and gait difficulties [5]. Symptoms typically occur during childhood or young adulthood, and early signs may include difficulty running or walking upstairs [6]. As the disease progresses, walking becomes increasingly impaired, and assistive devices may be required [6]. Indeed, many patients become wheelchair-bound because of severe spastic paraparesis and impaired ambulation [7]. Moreover, urinary urgency or incontinence can occur because of bladder dysfunction. Upper limb involvement is less common but can develop in some subtypes [6]. The rate of progression and severity of symptoms are highly variable, even within the same genetic type of HSP [2].

On February 20, 2023, we conducted a literature search of the most recent review articles through PubMed, Google Scholar, and the Index to Chiropractic Literature using the phrases “hereditary spastic paraplegia” and “chiropractor.” However, we did not identify any relevant studies.

Chiropractors are primary care clinicians who frequently evaluate patients for neuromuscular skeletal issues [8]. According to a large study on adverse events associated with chiropractic treatments, the incidence of severe adverse events associated with chiropractic spinal manipulative therapy was 0.21 per 100,000 treatments [9], making them an attractive option for the long-term management of HSP. The incidences are rib fractures occurring in geriatric women with osteoporosis [9], which does not apply to our case. Here, we report the case of a young female with HSP who underwent chiropractor rehabilitation, given the paucity of literature on the topic.

## Case presentation

A 13-year-old girl with a history of hereditary spastic lower body paralysis presented to a chiropractic clinic with chronic low back pain and lower extremity weakness. Her pain started three years ago and was rated 6/10 in the numeric pain score. The patient showed developmental delays but was otherwise an active, healthy teenager. When she was four years old, she was diagnosed with pelvic imbalance due to excessive muscular stiffness, spasms, and weakness in the legs.After first experiencing hip pain at nine years of age, her leg stiffness and weakness steadily worsened over the past few years to the point where she now requires a wheelchair for mobility and her mother’s assistance with bathing and toileting. Although she was able to walk short distances with leg braces, she suffered from frequent falls. Significant muscle spasms and pain in her lower back and legs required a prescription of non-steroidal anti-inflammatory drugs (NSAIDs) and baclofen for spasticity.

The patient had no history of surgeries. She denied any bladder or bowel incontinence but reported constipation and “spastic pain” in her thighs and calves. She also had a history of pressure sores on her feet due to deformities and poor mobility. Regarding her family history, her brother had HSP with severe lower limb weakness and frequent hospitalization (every six months), while her mother had been diagnosed with panic and mood disorders and had been under medication for seven years.

Over the previous 12 months, the patient’s legs had become weaker, and she could only pace for a few moments with support; as a result, she spent most of her time sitting down, and her back muscles became more painful. She also complained of tiredness after walking for 5 meters. Therefore, her mother sought chiropractic therapy for her to improve her strength and function.

**Figure 1:**
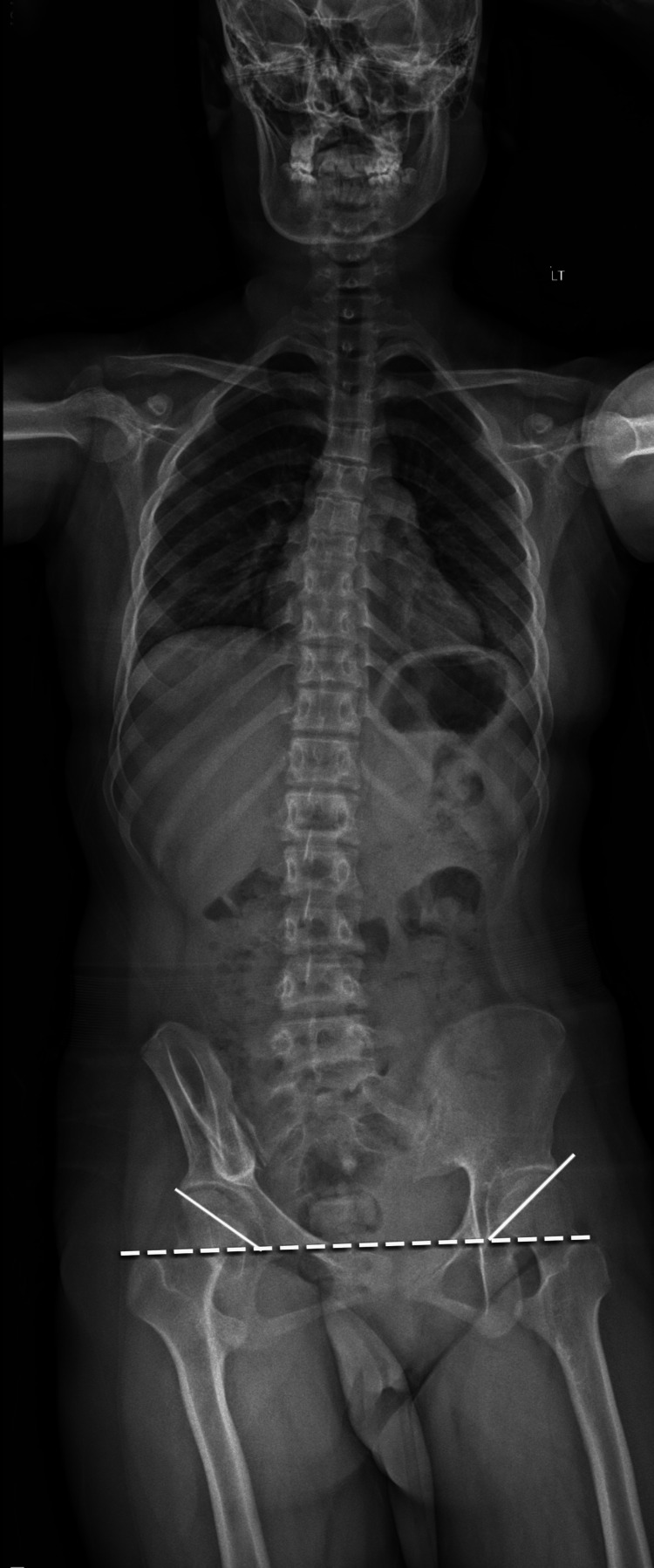
Standing full-spine radiograph The standing radiograph showed that the left acetabulum is superior to the right, with minor pelvic obliquity at 4°.

Physical examination revealed normal strength and tone of the upper extremities, while lower extremity examination revealed significant spasticity, hyperreflexia, and weakness, especially on the right side. The patient had ankle clonus and hyperactive patellar and Achilles tendon reflexes bilaterally. The strength was 3/5 for the hip flexors and extensors, 2/5 for the knee flexors and extensors, and 1/5 for the ankle dorsiflexors and plantar flexors. Spasticity had resulted in partially flexed legs, although the sensation from light touching remained intact. Her gait was significantly impaired due to lower extremity spastic weakness. Other aspects of the physical examination, including cardiovascular, pulmonary, and abdominal examinations, were within normal limits. Full-spine radiography revealed minor pelvic obliquity and borderline acetabular dysplasia in the right hip (Figure 1). The acetabular angle of Sharp on the superior/right and inferior/left sides was 45° and 36° (normal = 33º to 38º; acetabular dysplasia > 42º or ≥ 45º) [10,11]. In cases where the angle is between the upper border of the normal range, the terms “borderline” or “indeterminate” can be used [10,11].

**Figure 2:**
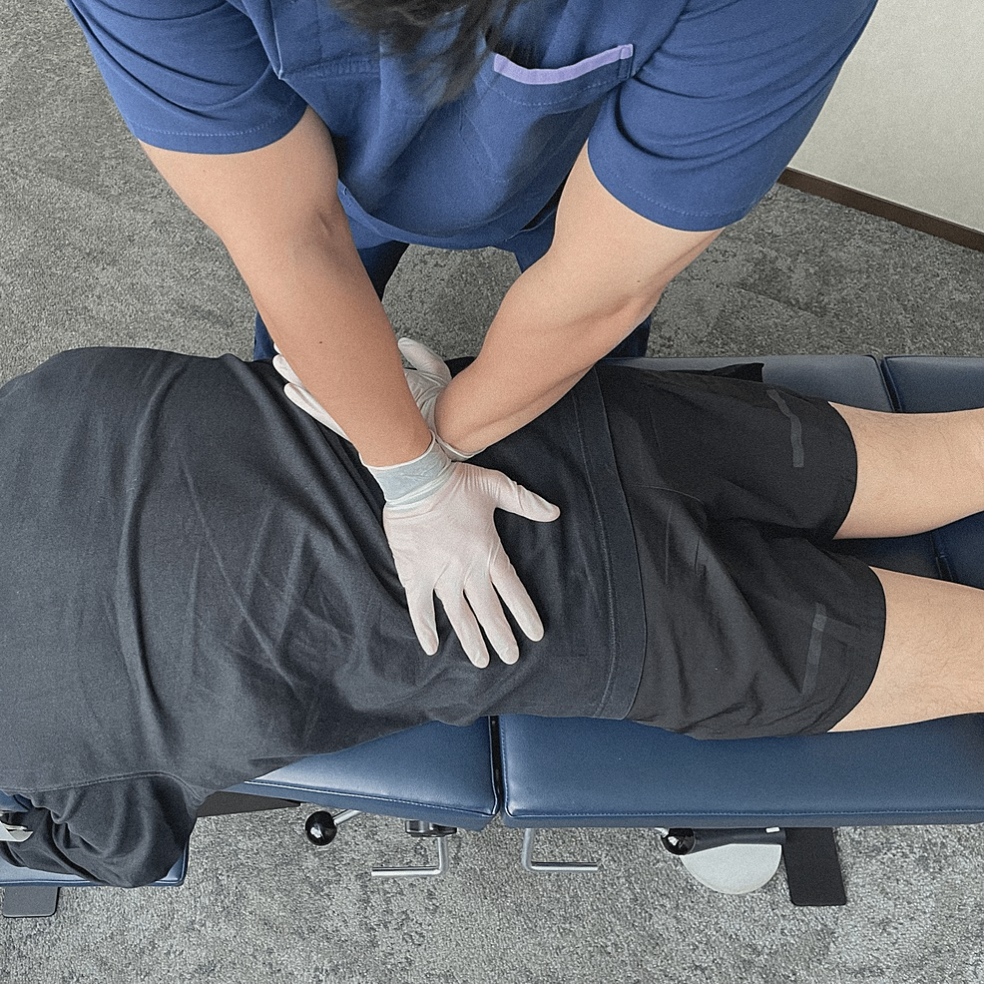
Demonstration of drop piece adjustments to the lumbopelvic spine Chiropractic spinal manipulative therapy (drop piece adjustment) to the lumbopelvic spine for relieving the pressure on nerve roots.

**Figure 3:**
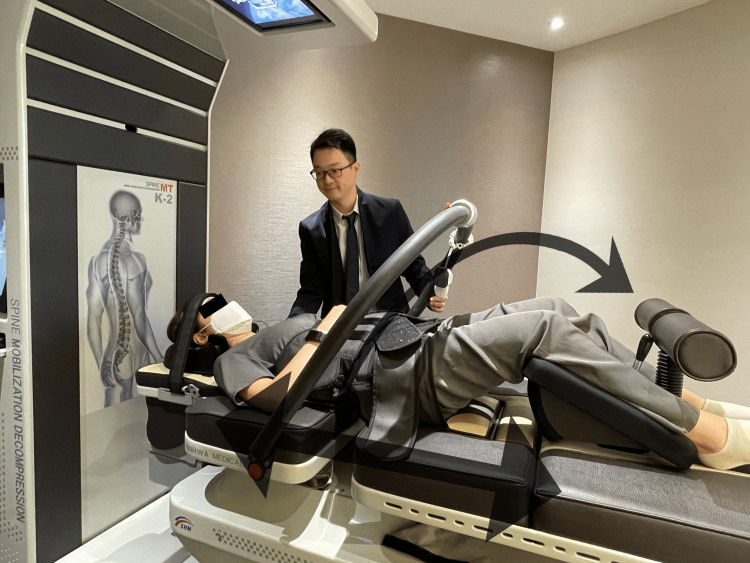
Demonstration of mechanical spinal distraction Mechanical spinal distraction (SpineMT, Korea) was applied to manage spinal deformity [12] and increase the range of motion of the pelvic and lower extremity joints.

Chiropractic treatment focuses on spinal manipulation, soft tissue release techniques, and rehabilitation exercises to help alleviate tension on the nerves and improve mobility and flexibility. Specific manipulation techniques, including drop piece adjustments (Figure 2) to the lumbar spine and pelvis to relieve pressure on nerve roots and mechanical spinal distraction (Figure 3), were applied to balance the pelvic floor and increase the range of motion of the pelvic and lower extremity joints.

**Figure 4:**
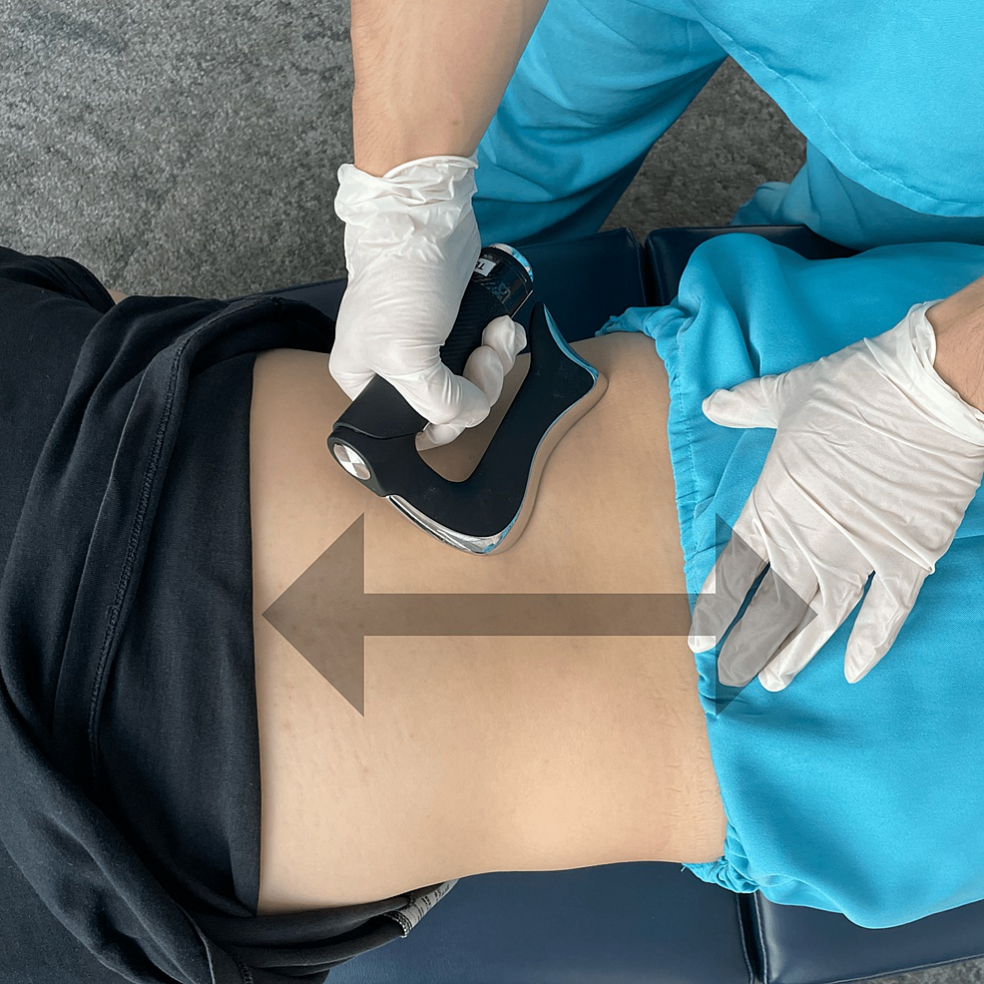
Demonstration of instrument-assisted soft tissue mobilization (IASTM) Scraping therapy (i.e., IASTM) is applied to the lower back muscle to alleviate muscular hypertonicity and spasm.

Instrument-assisted soft tissue mobilization (IASTM), shown in Figure 4, was also used to alleviate the soreness of muscular hypertonicity and spasm in the lower back, pelvis, hip, and lower limbs. Gait training with orthotics, stretching, and strengthening exercises for hamstrings and hip adductors were performed in the clinic and assigned as daily homework to improve the range of motion and gait pattern. Treatment sessions of 15-30 minutes were scheduled two to three times per week for the first month and then once per week for the next two months to observe improvement and modify the plan as needed. After the first month of treatment, the patient reported an improvement in quality of life from 56% to 64%. After eight weeks of therapy, her pain and spasms decreased by more than 50%, allowing her to discontinue the pain medication intake from daily to twice a week to manage minor symptoms. Her schedule was reduced to once a month for six months. She could walk farther and felt more stable on her feet. Incidentally, her bowel and bladder control also improved, resulting in a better quality of life with more independence and mobility (from 56% to 80%) at the nine-month re-evaluation.

## Discussion

Although there may be different treatment options available for HSP, the primary focus of therapies are on symptom management [13]. As there is no cure for HSP, physical therapy and stretching can help maintain mobility and range of motion. Medications such as baclofen, tizanidine, and benzodiazepines may reduce spasticity but can cause side effects such as drowsiness and weakness [13]. Botulinum toxin injections provide temporary relief from spasticity, but repeated injections may lead to immunity [13]. Surgical procedures are risky and offer few lasting benefits. Although these treatment options can be helpful, the progressive nature of HSP and its potential side effects highlight the need for safe alternative therapies. Although current medical techniques aid in the diagnosis and management of the disease, current symptomatic treatments fall short of expectations [1]. Chiropractic care shows promise in decreasing spasticity and improving mobility [14], although larger studies are needed.

Chiropractic therapy may improve HSP symptoms by reducing spinal nerve irritation and increasing joint mobility. Moreover, reducing inflammation and improving nerve function could help decrease excessive muscle tone and spasms. Studies on chiropractic treatment for spasticity resulting from multiple sclerosis and cerebral palsy suggest that it can decrease pain, improve range of motion, and increase walking speed [14,15]. Although the mechanisms are not fully understood, chiropractic manipulative therapy increases the cortical intrinsic inhibitory interactions in patients with chronic pain [16,17], normalizes muscle tone, and inhibits spasms [18]. Other studies have also shown that chiropractic adjustments can increase the dorsiflexor muscle strength and walking gait in stroke patients [19,20]. Pregnant women in their second trimester also experience more relaxation of the pelvic floor muscles after chiropractic treatment [21]. Moreover, scraping therapy has been found to increase the range of motion in stiff joints and muscles and improve the strength of the immune system [22]. As non-invasive therapies have minimal side effects [9], they can be used alongside other treatments or as a complement to traditional therapy for HSP, including for long-term management.

Studies have also shown that regular chiropractic treatment and home exercises could maintain the improvements in symptoms and function despite disease progression [23]. Monthly maintenance chiropractic care has also been reported to improve spinal deformity, which may benefit patients with spasticity and mobility disorders [24]. The current case suggests that chiropractic therapy, soft tissue techniques, and exercise decrease spasticity and improve mobility, which warrants further study in these complex conditions. Although HSP is a rare disease, more research, especially larger clinical trials, is recommended to fully understand the potential scope of chiropractic benefits and limitations for different types of spasticity and patient populations. Additional studies could help to determine the optimal dosage, length of treatment, and long-term outcomes. Chiropractic care can be integrated into a multidisciplinary approach for spasticity management, which will help to develop comprehensive plans tailored to a patient’s individual needs and goals.

## Conclusions

Chiropractic care may help reduce lower extremity spasticity and pain, improve strength and functionality, and prevent complications in conditions such as HSP. Spinal manipulation and soft tissue techniques can decrease nerve irritation and muscle tension, which may alleviate spasms and pain. Regular chiropractic treatment and home exercises could help maintain the improvements in symptoms and function, with the potential to decrease the reliance on medications and their associated side effects. Although larger controlled studies are needed, the potential benefits of chiropractic therapy for spasticity highlight its promise as a safe, conservative treatment option.

## References

[1] Meyyazhagan A, Orlacchio A (2022). Hereditary spastic paraplegia: an update. Int J Mol Sci.

[2] Panza E, Meyyazhagan A, Orlacchio A (2022). Hereditary spastic paraplegia: genetic heterogeneity and common pathways. Exp Neurol.

[3] Koh K, Ishiura H, Tsuji S, Takiyama Y (2018). JASPAC: Japan Spastic Paraplegia Research Consortium. Brain Sci.

[4] Hsu SL, Hsueh HW, Chen SY (2021). Clinical and genetic characterization of hereditary spastic paraplegia type 3A in Taiwan. Parkinsonism Relat Disord.

[5] Murala S, Nagarajan E, Bollu PC (2021). Hereditary spastic paraplegia. Neurol Sci.

[6] Fink JK (2006). Hereditary spastic paraplegia. Curr Neurol Neurosci Rep.

[7] Ebrahimi-Fakhari D, Teinert J, Behne R (2020). Defining the clinical, molecular and imaging spectrum of adaptor protein complex 4-associated hereditary spastic paraplegia. Brain.

[8] Chu EC, Trager RJ, Lee WT (2022). Use of thrust cervical spinal manipulative therapy for complicated neck pain: a cross-sectional survey of Asia-Pacific chiropractors. Cureus.

[9] Chu EC, Trager RJ, Lee LY, Niazi IK (2023). A retrospective analysis of the incidence of severe adverse events among recipients of chiropractic spinal manipulative therapy. Sci Rep.

[10] Mannava S, Geeslin AG, Frangiamore SJ, Cinque ME, Geeslin MG, Chahla J, Philippon MJ (2017). Comprehensive clinical evaluation of femoroacetabular impingement: part 2, plain radiography. Arthrosc Tech.

[11] Welton KL, Jesse MK, Kraeutler MJ, Garabekyan T, Mei-Dan O (2018). The anteroposterior pelvic radiograph: acetabular and femoral measurements and relation to hip pathologies. J Bone Joint Surg Am.

[12] Chu EC, Cheng HY, Huang K, Yao K, Zhao J (2023). Conservative management of low back pain and scoliosis in a patient with rheumatoid arthritis: eight years follow-up. Cureus.

[13] Bellofatto M, De Michele G, Iovino A, Filla A, Santorelli FM (2019). Management of hereditary spastic paraplegia: a systematic review of the literature. Front Neurol.

[14] Kachmar O, Kushnir A, Matiushenko O, Hasiuk M (2018). Influence of spinal manipulation on muscle spasticity and manual dexterity in participants with cerebral palsy: randomized controlled trial. J Chiropr Med.

[15] Kachmar O, Voloshyn T, Hordiyevych M (2016). Changes in muscle spasticity in patients with cerebral palsy after spinal manipulation: case series. J Chiropr Med.

[16] Haavik H, Kumari N, Holt K (2021). The contemporary model of vertebral column joint dysfunction and impact of high-velocity, low-amplitude controlled vertebral thrusts on neuromuscular function. Eur J Appl Physiol.

[17] Haavik H, Niazi IK, Holt K, Murphy B (2017). Effects of 12 weeks of chiropractic care on central integration of dual somatosensory input in chronic pain patients: a preliminary study. J Manipulative Physiol Ther.

[18] Chu EC, Wong AY, Lee LY (2021). Chiropractic care for low back pain, gait and posture in a patient with Parkinson's disease: a case report and brief review. AME Case Rep.

[19] Navid MS, Niazi IK, Lelic D (2021). Chiropractic spinal adjustment increases the cortical drive to the lower limb muscle in chronic stroke patients. Front Neurol.

[20] Holt K, Niazi IK, Amjad I (2021). The effects of 4 weeks of chiropractic spinal adjustments on motor function in people with stroke: a randomized controlled trial. Brain Sci.

[21] Haavik H, Murphy BA, Kruger J (2016). Effect of spinal manipulation on pelvic floor functional changes in pregnant and nonpregnant women: a preliminary study. J Manipulative Physiol Ther.

[22] Chu EC, Wong AY, Sim P, Krüger F (2021). Exploring scraping therapy: contemporary views on an ancient healing - a review. J Family Med Prim Care.

[23] Iben A, Lise H, Charlotte LY (2019). Chiropractic maintenance care - what's new? A systematic review of the literature. Chiropr Man Therap.

[24] Chu EC (2022). Reducing cervical retrolisthesis with long-term monthly chiropractic maintenance care: a case report. J Med Cases.

